# Toward an NGF-based therapy for Rett syndrome

**DOI:** 10.3389/fnins.2026.1800227

**Published:** 2026-04-24

**Authors:** Giulia Borgonovo, Alexia Tiberi, Simona Capsoni, Antonino Cattaneo

**Affiliations:** 1 Bio@SNS Laboratory of Biology, Scuola Normale Superiore, Pisa, Italy; 2 Section of Physiology, Department of Neuroscience and Rehabilitation, University of Ferrara, Ferrara, Italy; 3 European Brain Research Institute (EBRI), Fondazione Rita Levi-Montalcini, Rome, Italy

**Keywords:** intranasal treatment, microglia, mitochondria, nerve growth factor (NGF), Rett syndrome

## Abstract

Rett syndrome (RTT) is a severe neurodevelopmental disorder primarily caused by mutations in the MECP2 gene. Although recent therapeutic advances, such as the approval of Trofinetide, offer partial relief, no comprehensive curative treatment is currently available. Among the emerging strategies, nerve growth factor (NGF) has gained attention due to its neurotrophic and immunomodulatory properties. This review, in addition to discussing the key features of RTT and the role of growth factors, also highlights recent evidence supporting NGF-based strategies for RTT, focusing on two independent studies that tested intranasal administration of NGF-like molecules in Mecp2-mutant mice. Both recombinant human NGF (rhNGF) and a modified, “painless” variant (hNGFp) improved behavioral (cognitive and motor) symptoms. While rhNGF primarily restored mitochondrial function, hNGFp restored neuroinflammatory responses through microglial regulation. Despite differences in molecular mechanisms and dosages, both molecules demonstrated efficacy without adverse effects, especially when administered intranasally, preventively, and over longer periods. These findings suggest that NGF may act through dual mechanisms, by supporting energy homeostasis and regulating immune responses. The use of intranasal delivery further enhances translational potential by overcoming blood–brain barrier limitations. Together, these studies provide a strong rationale for pursuing NGF-based therapies in RTT and encourage further investigations to optimize dosing, timing, and safety in preclinical and clinical settings.

## 1 Introduction

**Figure 1:**
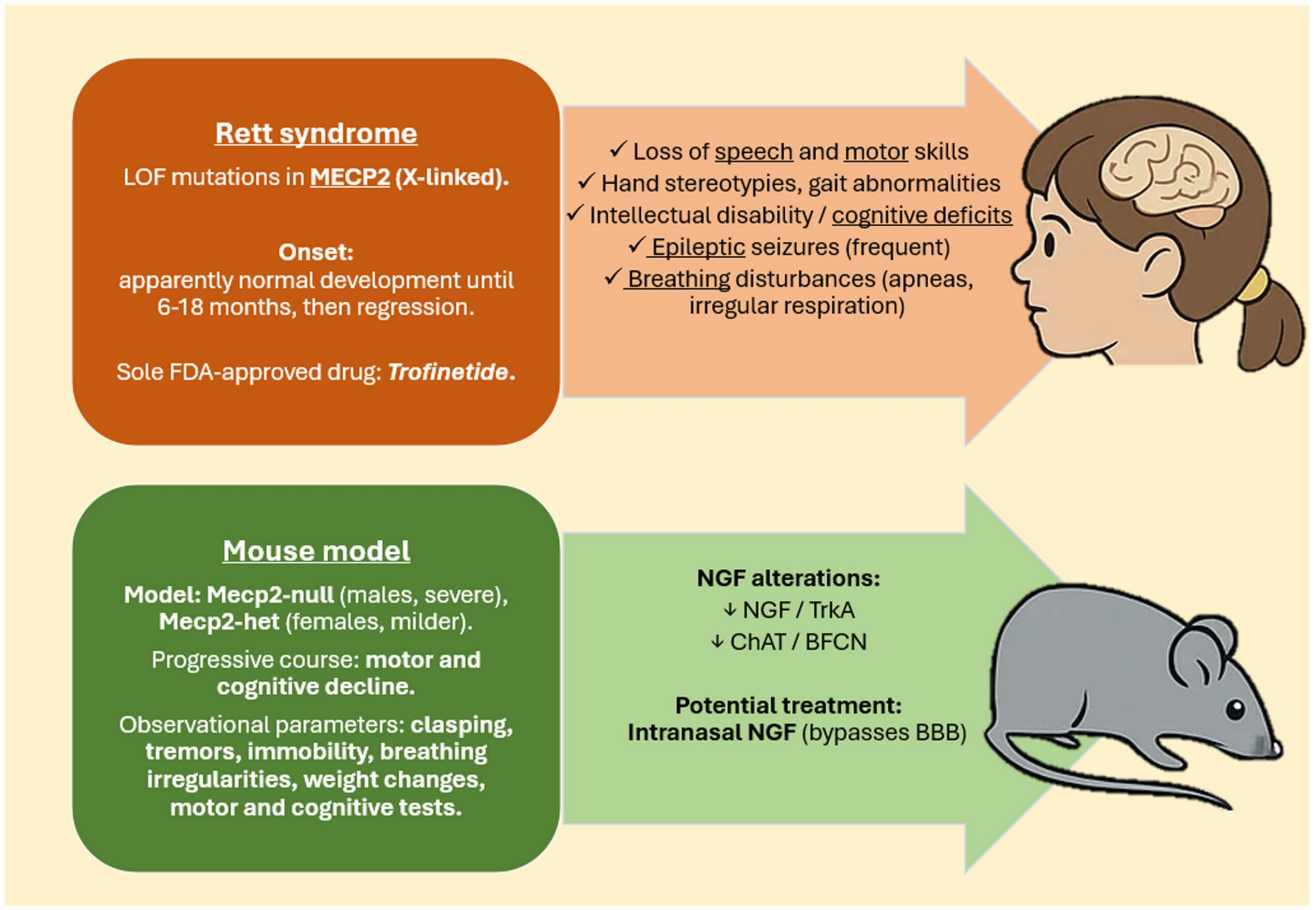
Illustration of Rett syndrome pathophysiology and experimental mouse model. Rett syndrome is an X-linked neurodevelopmental disorder caused by MECP2 loss-of-function (LOF) mutations, almost exclusively affecting females. After a period of apparently normal development, children undergo regression with motor and cognitive decline, seizures, breathing abnormalities, and loss of acquired skills. The only FDA-approved drug is Trofinetide. Mecp2-null and Mecp2-het mice recapitulate key clinical features, including clasping, tremors, immobility, irregular respiration, weight changes, and worsening condition. Among different abnormalities, the mouse model is also characterized by alterations in NGF/TrkA signaling and in basal forebrain cholinergic neurons, which suggest a role for neurotrophic pathways. Intranasal NGF, which bypasses the BBB, represents a potential therapeutic approach.

Rett syndrome (OMIM identifier #312750, RTT) is a rare severe neurological disorder that impairs cognitive and motor development and functionality. In the majority of cases (90–95%), individuals diagnosed with the classical form of RTT harbor loss-of-function mutations in the X-linked methyl-CpG binding protein 2 (MeCP2) gene, encoding for a ubiquitous protein abundantly produced in the brain (Amir et al., 1999). Due to MeCP2 chromosomal localization, RTT arises almost exclusively in females, with an incidence of approximately 1 in 10,000 live births. However, despite the common belief that the majority of males are fated to die *in utero* or in early infancy due to the hemizygous condition, a widespread comprehensive genomic testing in boys with neurodevelopmental problems has led to the definition of atypical RTT in males (Neul et al., 2019). To further complicate the clinical picture, random X chromosome inactivation results in somatic mosaicism in affected females, leading to variable expression of mutant and wild-type MeCP2 (Escamilla-Del-Arenal et al., 2011; Yang et al., 2011) (Figure 1).

The syndrome is not immediately apparent in the postnatal period, as affected individuals typically undergo a phase of apparently normal development lasting between 6 and 18 months (Hagberg et al., 1983; Chahrour et al., 2008). Following this period, mutations associated with RTT precipitate a critical phase of developmental regression, during which affected children lose previously acquired speech and motor skills, and exhibit stereotypic movements, gait disturbances, seizures, and cognitive impairment. Between 2 and 10 years old, patients reach a plateau phase, in which they may exhibit a slight improvement in cognitive engagement. Still, purposeful hand and body movements remain significantly impaired. Upon reaching middle age, female patients typically suffer from severe respiratory infections or cardiac complications, which lead to premature death (Anderson et al., 2014). From a preclinical perspective, rodents knock-out for MeCP2 or carrying mutations in the MeCP2 gene (Guy et al., 2001) are the most employed animal models to study RTT, as they faithfully recapitulate almost the entire range of the symptoms (Katz et al., 2012) (Figure 1).

To date, no definitive therapy is available for RTT. Until very recently, the main treatments were purely symptomatic, such as physical and orthopedic therapy, speech-language therapy, and nutritional support. Nevertheless, in March 2023, a major breakthrough was achieved when the U.S. Food and Drug Administration (FDA) approved an oral solution of *Trofinetide* (DAYBUE™) as the first treatment for RTT, in adults and pediatric patients of 2 years of age and older (Keam, 2023). This drug significantly improved symptomatology in 38% of patients.^1^ Still, the majority of treated patients experienced no symptom improvement or severe side effects (Hudu et al., 2023), highlighting the need for continued progress in both preclinical and clinical research in pursuit of a more comprehensive therapeutic solution.

## 2 Growth factors involvement in Rett syndrome

Neurodevelopment refers to that highly sensitive period during which a series of critical processes must unfold correctly to ensure the proper formation and function of the brain. Interference from environmental or genetic factors can lead to the disruption of such processes, which include neurogenesis, gliogenesis, cellular migration, cell differentiation, and synaptogenesis, ultimately leading to neurodevelopmental disorders (Reichard and Zimmer-Bensch, 2021). Central to the regulation of neurodevelopment are growth factors, a diverse group of molecules involved in cell proliferation, differentiation, and survival (Cameron et al., 1998; O’Kusky and Ye, 2012). Altered levels of growth factors during neurodevelopment can contribute to the onset of disorders like RTT (Galvez-Contreras et al., 2016, 2017), and represent a promising, druggable target for therapeutic intervention.

Direct regulation of growth factor levels by MeCP2 has been proven for the Brain Derived Neurotrophic Factor (BDNF) (Chen et al., 2003; Chang et al., 2006), which is a member of the *Neurotrophin family* generally known to be involved in synaptic maturation and plasticity (Carvalho et al., 2008). Overexpression of BDNF indeed restores locomotor activity levels in MeCP2 mutant mice and relieves some symptoms of the mutant phenotype (Chang et al., 2006). Furthermore, reduced BDNF expression in the brainstem correlates with respiratory dysfunction in MeCP2 mutant mice, and enhancing BDNF expression ameliorates respiratory symptoms (Ogier et al., 2007; Schmid et al., 2012; Deogracias et al., 2012). Unfortunately, the therapeutic utility of BDNF is hampered by its poor efficiency at crossing the blood–brain barrier (BBB) (Poduslo and Curran, 1996). For this reason, a clinical trial was conducted with Fingolimod, an orally active modulator of sphingosine-1-phosphate receptors known to elevate BDNF levels in MeCP2 mouse models. However, no conclusive results on its efficacy were obtained (Naegelin et al., 2021).

As mentioned above, the only available therapy for RTT indeed relies on the delivery of a synthetic analog of the bioactive tri-peptide N-terminal portion of the insulin-growth factor-1 (IGF-1): *Trofinetide*. IGF-1, similarly to BDNF, promotes neuronal survival, synaptogenesis, and neuronal plasticity (Castro et al., 2014), but has the incredible advantage of being capable of crossing the BBB, particularly in its tripeptide form (Baker et al., 2005).

Recent research, including work from the authors of the present review, have instead focused the attention on the therapeutic potential of another growth factor of the neurotrophin family—the prototypical growth factor discovered by the Nobel Laureate Rita Levi-Montalcini in the 1950s—the nerve growth factor (NGF) (Levi-Montalcini, 1952).

### 2.1 NGF dysfunction in Rett syndrome

NGF stands out as a key neurotrophic factor essential for a wide range of physiological processes. It is the first discovered and best characterized neurotrophic factor essential for the growth, differentiation, and survival of sympathetic and sensory neurons, especially during development (Levi-Montalcini, 1966), and cholinergic neurons also during adulthood (Hefti, 1994; Mufson et al., 1999). Beyond these important functions, NGF also regulates nociception in adulthood by sensitizing peripheral nociceptors, thereby enhancing pain perception, particularly during tissue inflammation (Lewin and Mendell, 1993; Lewin et al., 1993; Chuang et al., 2001; Watanabe et al., 2008; Anand et al., 1996). Moreover, NGF also influences cells in the immune system and brain regions involved in neuroendocrine functions (reviewed in Fiore et al., 2009).

Given its pleiotropic nature, NGF holds promising therapeutic potential first and foremost because of its well-known neurotrophic and neuroprotective properties. However, this molecule is also of particular interest specifically for RTT, as multiple reports have highlighted its involvement in the disease.

Indeed, lower NGF plasma levels have been detected in RTT patients with prolonged cardiac QT intervals compared to those with normal QT intervals, which could possibly explain the cardiac arrhythmias seen in RTT (Guideri et al., 2004), since NGF production by heart cells promotes proper cardiac function (Lockhart et al., 1997). Related to that, no significant differences in NGF serum levels were found between RTT patients and controls, though NGF levels were reduced in the cerebrospinal fluid of RTT patients (Lappalainen et al., 1996).

Moreover, immunohistochemistry in RTT patients’ post-mortem brain tissue showed a lower expression of NGF and Tropomyosin receptor kinase A (TrkA, the high-affinity receptor of NGF), implicating NGF signaling pathway deficiency in impaired neuronal development in RTT (Lipani et al., 2000). However, in certain regions, areas of astrogliosis have been found to overlap with NGF-positive zones, suggesting a possible causal relationship between astrocyte activation and the production of this neurotrophic factor (Lipani et al., 2000). Indeed, as the authors of this work speculatively suggested, in RTT, the high frequency of epileptic seizures may act as an injurious stimulus capable of activating astrocytes and microglia, leading to an inflammatory response and increased NGF expression (Strauss et al., 1994). This mechanism could represent an adaptive response of the brain and may partly explain the mild clinical improvements observed in some patients during the plateau phase of the disease.

One main recognized role of NGF during both neurodevelopment and adulthood is to promote basal forebrain cholinergic neurons (BFCNs) survival and differentiation (Perry, 1990). Two decades ago, impairments in BFCNs were identified in RTT, potentially contributing to cognitive dysfunctions. These studies showed decreased choline acetyltransferase (ChAT) function in many cortical and subcortical regions of RTT-affected girls (Wenk et al., 1993; Wenk and Hauss-Wegrzyniak, 1999), and in particular in BFCNs (Wenk et al., 1993; Wenk and Hauss-Wegrzyniak, 1999). In this framework, recent animal studies further emphasize the involvement of BFCNs in RTT. Ballinger and colleagues used the Cre-lox system to delete MeCP2 from cholinergic neurons, resulting in recognition memory impairments and cortical neuron dysfunctions, which were restored by treatment with the cholinesterase inhibitor *Donepezil*, thus by increasing acetylcholine availability (Ballinger et al., 2019). Conversely, Zhou and colleagues demonstrated that selectively re-expressing MeCP2 in BFCNs of global MeCP2-null mice rescued locomotion, grip strength, and anxiety-like behavior, indicating a significant role of BFCNs in RTT (Zhou et al., 2017). Hence, the fact that BFCNs utterly rely on NGF for their survival and function (DiStefano et al., 1992) raises the possibility that reduced levels of NGF or NGF receptors in the brains of individuals with RTT may underlie the observed cholinergic deficits.

All together, these findings suggest that the NGF signaling pathway could play a role in this neurodevelopmental disorder, still needed to be properly explored.

## 3 NGF limitations in clinical practice

Following its discovery, NGF’s potent neurotrophic action quickly positioned it as a potential therapeutic agent for a wide array of neurological pathologies (including Alzheimer’s, Parkinson’s disease, brain trauma, and ocular disorders) and other CNS-associated conditions (such as diabetic polyneuropathy and human immunodeficiency virus (HIV)-related peripheral neuropathies), regardless of whether these diseases exhibit neurotrophic system perturbations (reviewed in Aloe et al., 2012). As a matter of fact, numerous NGF clinical trials have targeted these diseases, but many were either ineffective or significantly limited despite encouraging preliminary results. The main reasons for these clinical failures for CNS pathologies are related to two significant issues: (i) poor NGF brain delivery and biodistribution due to its inability to cross the BBB (Pan et al., 1998; Poduslo and Curran, 1996), and (ii) NGF-induced pain from inflammatory responses and nociceptive system hyperactivation, considered as a non-neglectable side effect (Lewin and Mendell, 1993; Lewin et al., 1993; Chuang et al., 2001).

To overcome the first hurdle (inability to cross the BBB), two strategies have been developed so far: the use of small molecules activating NGF signaling (NGF mimetics) and the delivery of NGF via the intranasal route.

## 4 NGF mimetics in Rett syndrome

A recent study introduced a novel approach to investigating the neurotrophin pathway in RTT, which is also marked by chronic systemic oxidative stress (OS) and inflammation. The p75 neurotrophin receptor (p75NTR), a shared receptor for NGF and other neurotrophins, is known to play a key role in regulating pathways linked to OS and inflammation (Kraemer et al., 2014). To explore this connection, Segatto’s group employed primary fibroblast cultures derived from RTT patients and healthy donors as experimental models (Varone et al., 2024). In RTT fibroblasts, both NGF and its receptor p75NTR were significantly reduced at mRNA and protein levels, indicating a downregulation of the NGF/p75NTR pathway. Importantly, they tested LM11A-31, a molecule that binds to the loop 1 domain of p75NTR, the same region where NGF interacts. Unlike traditional agonists or antagonists that broadly stimulate or block receptor functions, LM11A-31 acts as a modulator of p75NTR signaling, by selectively enhancing pro-survival signaling via the PI3K/PKB (Akt) pathway, promoting cell survival and growth while suppressing degenerative pathways associated with neurodegeneration and apoptosis (Massa et al., 2006). The study demonstrated that LM11A-31 effectively mitigates OS and inflammatory alterations in RTT fibroblasts. Interestingly, LM11A-31 has progressed to clinical trials for mild to moderate Alzheimer’s disease, showing a good safety profile and some efficacy in targeting key pathological features (Shanks et al., 2024), thus setting the basis for a possible clinical trial in RTT. From a central nervous system perspective, this study provides valuable but indirect evidence, as the observations may not fully recapitulate the complexity of neurotrophin dynamics in brain cell types such as neurons, astrocytes, oligodendrocytes, or microglia; nevertheless, the results remain promising and support further investigation in more physiologically relevant animal models of RTT.

During the same period, another study highlighted a correlation between the modulation of the neurotrophic pathway and its potential therapeutic effect for RTT (Fazzari et al., 2024). Membrane receptors, such as neurotrophin Trk receptors, are influenced by the surrounding lipid microenvironment, with the ganglioside GM1 (monosialotetrahexosylganglioside) playing a pivotal role in regulating these functions (Pitto et al., 1998; Ledeen and Wu, 2008). Notably, reduced levels of GD1a, the metabolic precursor of GM1, have been observed in both Mecp2-null mice and RTT patients (Lekman et al., 1991, 1999). In this context, Fazzari and colleagues investigated GM1-OS, a bioactive derivative known for its neuroprotective and neurotrophic effects, which interacts with neurotrophin receptors on the cell surface, such as TrkA (Chiricozzi et al., 2017, 2019). *In vitro*, GM1-OS was shown to restore synaptogenesis and counteract mitochondrial oxidative stress. *In vivo*, GM1-OS administration improved RTT symptoms and mitigated motor deficits (Fazzari et al., 2024). These findings suggest that GM1-OS alleviates RTT phenotypes by activating Trk receptor activity on the plasma membrane.

## 5 Intranasal administration of NGF and an optimized NGF variant in RTT mouse models

Another promising strategy to deliver NGF safely to the brain is the non-invasive olfactory route (Malerba et al., 2011; Thorne and Frey, 2001; Gomez et al., 2012). In fact, the efficacy of intranasal delivery when administering NGF and its derivatives to the brain was recognized by the late 1990s. A notable preliminary study on the biodistribution of radiolabeled NGF (^125I^NGF) demonstrated more efficient brain uptake via intranasal administration compared to intravenous administration in rats. Following the olfactory route, ^125I^NGF was detected in the olfactory bulb, cerebrum, and brainstem, marking the first evidence of effective non-invasive delivery of NGF to the brain (Frey et al., 1997).

As described by Thorne and Frey (2001), intranasal administration can occur through three mechanisms: (i) internalization into primary neurons of the olfactory epithelium with subsequent intracellular transport to the olfactory bulb, from where it reaches other CNS areas, (ii) absorption across the nasal epithelium to the submucosa, providing direct access to the CSF and allowing extracellular transport within perineuronal channels into the CNS or (iii) access to nasal regions with lower vascular density and permeability, thus enhancing drug access to extracellular pathways associated with cranial nerves (Thorne et al., 1995; Kumar et al., 2016). Specifically, given the rapid appearance of ^125I^NGF in the brain 20 to 60 min post-intranasal administration and the observed linear relationship between the administered dose and resulting brain concentrations, it is hypothesized that ^125I^NGF is transported through the extracellular pathway, which is faster than the intracellular route.

To date, two different human recombinant NGF molecules have been tested intranasally in mouse models of Rett syndrome, both conducted between 2024 and 2025, one by Capsoni’s group (Tiberi et al., 2024; Scuola Normale Superiore, Pisa, Italy), testing the so-called “painless” NGF (Capsoni et al., 2012) and the other by Landsberger’s group (Pozzer et al., 2025; IRCCS San Raffaele Scientific Institute, Milan, Italy), investigating wild type NGF. Both studies reported positive results from behavioral, cellular, and molecular analyses of mouse models. The remarkable similarity of the design of the two studies, which is very rare in preclinical studies, is a unique opportunity for a thorough comparison of the experimental approaches to identify commonalities and differences, and to examine variations between the studies, with the goal of refining and optimizing an NGF-based therapy for RTT.

### 5.1 The NGF molecule

The first aspect worth emphasizing is the use of distinct NGF molecules in the two studies, which, while similar in nature, exhibit notable differences in terms of signalling properties.

Pozzer and colleagues used a GMP-grade recombinant human NGF (rhNGF, Dompé Farmaceutici, Milan, Italy), the first topical biotech drug currently approved for treating neurotrophic keratitis, a rare degenerative corneal disease characterized by loss of corneal sensitivity (Roszkowska et al., 2024). Furthermore, intranasal rhNGF has been evaluated in two clinical trials, both of which have shown positive results thus far, one related to traumatic brain injury [three children affected by unresponsive wakefulness syndrome treated with rhNGF showed clinical conditions improved with no side effects (Chiaretti et al., 2017; Gatto et al., 2023; Curatola et al., 2023)], and the second related to Dry eye disease [they evaluated safety and efficacy of rhNGF eye drops in patients, obtaining significant improvement in symptoms (Sacchetti et al., 2020)]. Therefore, given these considerations, rhNGF stands as an excellent clinical candidate for Rett syndrome.

On the other hand, Tiberi et al. utilized a recombinant human NGF molecule referred to as “painless” (hNGFp), which is similar to rhNGF with the exception of two single amino acid mutations: one from proline 61 to serine allowing the detection of the exogenous human NGF against the endogenous one (P61S), and a second mutation localized in position 100, substituting one arginine with a glutamic acid (R100E) (Covaceuszach et al., 2010). This modification was inspired by the human genetic disease Hereditary Sensory Autonomic Neuropathy Type V (HSAN-V), whose main symptom is a congenital, chronic insensitivity to pain due to the mutation R100W in the NGF gene, while not showing cognitive deficits (Einarsdottir et al., 2004). The rationale for developing this molecule stems from the fact that NGF *per se* is known to induce pain during administration (Petty et al., 1994; Apfel, 2002): tests involving various acute hyperalgesia assays (such as mechanical or thermal hyperalgesia after intraplantar injection, and orofacial pain after nasal delivery), as well as chronic sensitization assays, demonstrated that hNGFp induces a pro-nociceptive response that is at least tenfold lower than that induced by the same dose of native NGF, while preserving the same neurotrophic activity (Capsoni et al., 2012; Malerba et al., 2015; Capsoni et al., 2017). In addition to its extensive validation in many preclinical studies (Refs), the GMP-grade hNGFp protein is being clinically tested in man in the ocular drop formulation for an ophtalmic therapeutic indication (NCT05733573).

These characteristics are of great interest when approaching a clinical application. In fact, NGF is one of the molecules released directly at the site of peripheral inflammation, and its exogenous administration causes pain, such as in peripheral injections in clinical trials. For instance, a Phase III trial in the early 2000s assessed subcutaneous NGF administration three times a week in diabetic polyneuropathy patients. Although the trial reported significant improvements in neuropathic symptoms after 12 months, dose-dependent hyperalgesia was noted (including back pain, injection site hyperalgesia, arthralgia, and severe myalgia) (Apfel et al., 2000, 2002). Similarly, a subcutaneous NGF trial for HIV-associated peripheral nerve complications reported patients experiencing pain at the injection site (McArthur et al., 2000). These severe pain side-effects caused the interruption of the clinical development of hNGF by systemic administration.

Pozzer and colleagues also evaluated the potential pain-inducing effects of rhNGF using the cold plate test. Their findings showed that WT mice treated with rhNGF did not exhibit increased pain sensitivity compared to untreated WT mice. However, it is important to note that intranasal administration bypasses systemic circulation, preventing NGF from sensitizing peripheral skin nociceptors. Instead, NGF may directly induce pain at the level of the olfactory mucosa by activating trigeminal nerve endings (Capsoni et al., 2017). This possibility was not assessed in the study, which, for instance, did not include a Von Frey test to evaluate sensitization in the olfactory region (Bonomini et al., 2023; Shinoda et al., 2011). This is particularly relevant in cases where patients may experience distress due to pain but are unable to effectively communicate it to their caregivers (Symons et al., 2013; O’Leary et al., 2017; Fabio et al., 2022; Byiers et al., 2023).

Another critical distinction to highlight when developing NGF-based therapies is the molecular mechanism underlying NGF signaling activation of different NGF-derivatives. As mentioned above, NGF interacts with two primary receptors: the high-affinity TrkA receptor, which promotes cell survival and differentiation, and the low-affinity p75NTR receptor, which plays a role in TrkA pathway regulation but, when activated independently, can induce apoptosis The rhNGF is expected to activate both pathways. In contrast, hNGFp, due to the R100E mutation, is a TrkA-biased molecule, favoring survival and differentiation signaling while completely sparing the p75NTR pathway (Capsoni et al., 2011).

Despite these molecular differences, both rhNGF and hNGFp retain their neurotrophic activity. Thus, while there are mechanistic distinctions between the two forms of NGF, these do not diminish NGF’s potent neuroprotective efficacy. Moreover, both studies (as well as others) have consistently demonstrated that NGF administration in wild-type models produced no adverse effects, further supporting its safety profile for therapeutic use.

### 5.2 The genetic background and sex of mice

Both studies utilized the Mecp2^tm1.1Bird^ mouse strain; however, there is a notable difference in the genetic background of the animals. In Tiberi et al. study, animals were maintained on the C57BL/6 background, whereas Pozzer et al. used a CD1 background, possibly due to considerations related to maternal care (not specified). This distinction is critical to highlight, as it is increasingly recognized that varying genetic backgrounds can lead to divergent outcomes, particularly in behavioral studies (Sultana et al., 2019). Nevertheless, the fact that both studies observed positive behavioral effects (as described below) underscores the robustness and potential translational value of the treatment, which is promising for future clinical applications.

Analysis of both sexes was included in Pozzer et al. study, despite the fact that in RTT research, male mice are often favored due to their significantly more severe phenotype compared to females (Ribeiro and MacDonald, 2020). In contrast, Tiberi and colleagues did not include a male cohort, as they prioritized a phenotype more closely aligned with that observed in humans.

### 5.3 Administration route, timing and dosage

As first approach, Pozzer and colleagues explored the intraventricular route, administering 270 μg/mL (approximately 90 μg/kg) into the right lateral ventricle, and assessed biodistribution over the following 48 h using ELISA. After 24 h, NGF was detected in the hippocampus, prefrontal cortex, striatum, brainstem, and cerebellum in both hemispheres. However, NGF concentrations declined sharply after this period.

Also Capsoni’s group, in a previous work, investigated a direct NGF delivery method in a different experimental model, the 5xFAD mouse model of Alzheimer’s disease, which they have studied extensively in relation to hNGFp (Capsoni et al., 2017). They tested intraparenchymal delivery via a mini-osmotic pump, targeting the nucleus basalis of Meynert (NBM) in 3-month-old 5xFAD mice, aiming to replicate current NGF clinical trial approaches. The findings revealed that localized delivery near the NBM was insufficient for hNGFp to exert a widespread neuroprotective or anti-amyloidogenic effect, which instead requires broader distribution within the brain – something that can be achieved through intranasal administration.

Indeed, due to the challenge of NGF crossing the BBB, and the invasiveness of the intracerebral ventricular route and intraparenchymal delivery, as anticipated, both studies described in this review employed intranasal delivery of the NGF molecule, a method previously demonstrated to be highly effective in distributing NGF throughout the brain within hours (Rosa et al., 2005; Manni et al., 2021; Capsoni et al., 2017).

In Tiberi et al. study, the intranasal delivery route was applied to two cohorts of female animals, with treatments administered three times per week (every 2 days). Two distinct treatment groups were established: the first one began at postnatal day 60 (P60), prior to the onset of symptoms, and continued until the animals were humanely sacrificed (max around P250), with the goal of evaluating preventive effects (long-term treatment). The second group received treatment starting when hindlimb clasping reached a score of 1 (as defined below), approximately at 3 months of age (P90), and this treatment lasted for 1 month, aimed at assessing therapeutic efficacy (short-term treatment).

In contrast, Pozzer and colleagues’ study administered daily treatments for 1 month, with male mice treated either from P30 to P60 or from P20 to P50, and female mice treated from P60 to P110.

This highlights a notable difference in the timing of administration. Since Tiberi and colleagues excluded male mice from their analysis, this comparison focuses solely on the female cohort. In Pozzer and colleagues’ study, the treatment in females is meant to be preventive, as it commenced at P60, before the onset of symptoms, which typically occurs around P90, and continued for 50 days. In comparison, Tiberi and colleagues’ short-term treatment regimen began at P90 and concluded at P120. This overlap provides a meaningful window for comparing molecular and cellular data between similarly age-matched mice across the two studies.

In preclinical studies, determining the appropriate dosage is crucial, not only for safety concerns but also for reducing manufacturing costs. Pozzer et al’s intranasal administration employed a dose of 2.1 mg/mL (with a total of 20 μL administered, equivalent to 4.2 mg/kg). In contrast, Tiberi et al. used a significantly lower dose of 0.54 μg/kg, based on the findings of (Capsoni et al., 2017), where this was the highest dose that did not induce pain in the orofacial region.

Despite the 8000 fold difference in dosing between rhNGF and hNGFp, both studies demonstrated the widespread biodistribution of NGF across multiple brain regions. In Pozzer and colleagues’ study, cortical NGF levels were measured at an average of 480 pg./mg of protein extract 3 h after injection, decreasing to 440 pg./mg 6 h later, and further dropping to 390 pg./mg at 16 h, thereby restoring physiological levels of 370 pg./mg. However, it is important to note that the authors did not employ a method to distinguish exogenous rhNGF from endogenous NGF produced within the brain.

In Tiberi et al. study, hNGFp biodistribution was not assessed for its detection, and this may be a limitation of the project. The authors however relied on previous the results obtained on the hNGFp detection through the P61S recognition tagging mutation WT mice (Capsoni et al., 2017). Indeed, after a single intranasal administration, the mutein was first observed in the olfactory bulb and trigeminal ganglion. Six to 24 h post-administration, hNGFp was found in the hippocampus, cerebral cortex, and cerebellum, with concentrations ranging from 500 to 1,000 pg./mg of protein. Notably, no hNGFp was detected in the blood serum at any time point, indicating that intranasal delivery leads to widespread distribution in key brain regions involved in neuropathology, such as the cortex and hippocampus, while remaining undetectable in the systemic circulation.

To further advance understanding of NGF’s theraputic effects in RTT, it would be beneficial to conduct studies using a dosage of rhNGF comparable to the 4 orders of magnitude lower dose of hNGFp, which has already demonstrated efficacy. Positive results from such studies could validate NGF-based therapies while achieving the same therapeutic effects with a dose that is 1,000 times lower.

## 6 Behavioral outcome

One of the primary methods for assessing animal welfare in RTT research involves behavioral analysis across various dimensions. This includes monitoring behavioral parameters such as hindlimb clasping, gait, breathing, tremors, immobility, and general condition, as well as conducting cognitive-related tests (like NOR or Marble test) and motor function tests (such as the rotarod, the beam test or pole test).

### 6.1 Behavioral parameters and general condition

Pozzer and colleagues extensively studied the behavioral aspects in male mice, both in the P30-P60 treatment (*later treatment*) and in the P20-P50 treatment (*early treatment*).

Concerning the later treatment, they revealed a typical decline in general health for untreated KO mice. However, rhNGF-treated mice showed a modest yet significant reduction in cumulative severity scores, starting at the almost end of the treatment, around day 26 of treatment, indicating that rhNGF delayed symptom progression. Improvements in mobility, hindlimb clasping, and tremors were observed, though overall condition remained stable. Additionally, rhNGF prevented KO mice from gaining weight over time, though it did not induce weight loss in either genotype.

They also administered intranasal treatments to males from P20 to P50, prior to symptom onset (early treatment). Twenty days after treatment began, they observed a marked improvement in general well-being. Treated KO animals also showed no tendency to gain weight, unlike their untreated WT littermates. Further analysis suggested that treatment during P20 to P50 was more effective in improving general health, mobility, and tremors than treatment from P30 to P60.

Regarding female mice (heterozygous for the mutation), which were treated from P60 to P110, no significant improvement was found in the cumulative severity score. However, secondary phenotype scores indicated a slight worsening in general condition, mobility, and tremors for untreated animals, with minor effects on weight gain. This indicates mild results for females treated with rhNGF.

As mentioned above, Tiberi and colleagues’ study is lacking a male cohort, thus the only comparison worth to be highlighted is in female heterozygous mice. Short-term hNGFp treatment (from P30 to P60) yielded moderate improvements, particularly in gait and clasping scores, while immobility and tremors remained unaffected. No significant changes were detected in body or brain weight. Overall, this treatment showed similar outcomes to those observed with rhNGF. In contrast, long-term hNGFp treatment (from P60 until the endpoint) significantly improved a variety of behavioral parameters, including hindlimb clasping, gait, respiration, tremors, immobility, and the overall phenotypic score.

Collectively, these findings for both rhNGF and the low dose of hNGFp suggest that, regardless of genotype or sex, longer preventive treatments are likely more effective than shorter, curative interventions in alleviating disease symptoms. This suggests that the presence of NGF in the brain may foster a neuroprotective (and/or glioprotective) environment, which could help prevent or at least mitigate the severity of the incoming symptoms.

### 6.2 Motor tests

One of the primary symptoms of Rett syndrome is severe motor impairment, which typically begins as early as the second year of life and worsens into adulthood. While some individuals retain the ability to walk, others develop bradykinesia and increased muscle tone, eventually losing the ability to walk due to difficulty maintaining upright postures (Nomura and Segawa, 1992; Brunetti and Lumsden, 2020). As a result, preclinical RTT studies also focus on assessing motor improvements in treated animals.

In the later treatment study on male mice (P30-P60) by Pozzer and colleagues, motor skills were evaluated using the Pole test which assesses motor coordination and balance in rodents by measuring the time taken to descend a vertical pole. No significant improvement was seen in the time it took mice to descend from the pole at P50. However, a longitudinal analysis on the behavior of these mice (indicated as the difference in performance between the baseline condition at P30 and that one at P50) showed that untreated KO mice experienced a marked decline in motor abilities, while the rhNGF-treated group showed a significantly better outcome. In contrast, early treatment produced a notable improvement in motor function by P50, as indicated again by the Pole test. To further assess motor benefits from early treatment, the Beam Walk test, which measures fine motor coordination and balance, was introduced. At P45, untreated KO mice took significantly longer to traverse the elevated beam compared to WT mice, whereas treated mice showed no such deficits. Similarly, rhNGF treatment nearly reversed the motor deficits in untreated female HET mice during the Beam Walk test, leading to an almost complete recovery.

Comparable results were seen with long-term hNGFp treatment by Tiberi and colleagues (P60 to the humane endpoint). At around P150 (5 months of age), female mice were tested on the Beam Walk test: hNGFp treatment improved both the distance traveled and the overall score, although treated mice still took longer to complete the task than WT controls. Notably, using the rotarod test, no improvements in motor coordination were observed following the shorter treatment period (P90-P120).

These findings reinforce the notion that initiating treatment before the onset of symptoms is more effective than starting after symptoms have appeared, also from a motor point of view. Notably, the Beam Walk test proved to be more sensitive than the Pole test in detecting fine motor impairments and evaluating treatment efficacy in motor disorders, and both neurotrophins showed neurotrophic potential in restoring motor function, with earlier and prolonged treatment yielding greater benefits.

### 6.3 Cognitive tests

Lastly, from a behavioral perspective, RTT is associated with intellectual disability, making the evaluation of cognitive functions in mouse models crucial for assessing the efficacy of potential treatments (Neul and Zoghbi, 2004; Berger-Sweeney, 2011). Pozzer and colleagues, in contrast to Tiberi et al., placed a significant emphasis on the cognitive aspect.

Using the Novel Object Recognition (NOR) test, they evaluated mice treated from P30 to P60 (later treatment) at P48. To ensure that the reduced mobility in untreated KO mice did not skew the results, the Open Field test was conducted beforehand. Remarkably, rhNGF treatment restored short-term memory deficits in KO mice, highlighting its cognitive benefits. They also utilized the Marble Burying test to examine environment-directed exploratory behavior, where untreated KO mice exhibited expected impairments, which were fully reversed after 10 and 25 days of treatment.

Further confirmation of rhNGF’s positive effects on memory came from the NOR test in animals treated from P20 to P50 (early treatment), where memory deficits seen in untreated KO mice were no longer present in treated animals, with a trend toward improvement. As before, the Open Field test verified that mobility deficits did not influence the results. The Marble Burying test once again demonstrated a significant improvement in environmental interaction at the end of the treatment, though no benefit was observed after just 10 days.

Female mice treated with rhNGF no longer exhibited the short-term memory deficits observed in untreated controls, confirming rhNGF’s positive effect on cognitive function. The Marble Burying test, however, showed no significant differences between untreated WT and HET mice.

### 6.4 Breathing test

Breathing difficulties are among the most severe symptoms of RTT. In mouse models, respiratory function can be assessed by observing how often the animals gasp or pant within a certain period when standing still (part of the observational behavioral parameters) (Guy et al., 2007). However, using automated tools to measure respiratory parameters can provide more detailed insights.

Pozzer and colleagues analyzed this respiratory aspect in KO mice, which partially replicate the breathing difficulties seen in RTT patients. They examined the effects of rhNGF on respiratory function using whole-body plethysmography. The analysis showed that rhNGF treatment significantly improved respiratory abnormalities, such as inspiratory times and the frequency of apneas, after 10 days of treatment. These improvements were not maintained after 24 days of treatment.

### 6.5 Survival curve

Despite these important benefits, rhNGF did not increase the life expectancy of the KO mice (not tested in HET mice), suggesting that the treatment protocol could be refined for this mouse model. Instead, Tiberi et al. found that hNGFp treatment from P30 up to the humane endpoint significantly increased the lifespan of HET female animals, with an increase in median survival of 30%.

## 7 Cellular and molecular underpinnings

Treatment with NGF, whether using rhNGF or hNGFp, consistently led to an overall improvement in the well-being of treated KO or HET animals, regardless of the differences in dosage or timing.

To gain a deeper understanding of the cellular and molecular mechanisms of NGF in these model animals, the two research groups employed two distinct approaches. Tiberi and colleagues focused on the inflammatory aspect of the disease, while Pozzer and colleagues concentrated on cellular respiration.

The latter used bulk RNA sequencing on the cortices of WT and KO mice, both treated and untreated with rhNGF. As expected, they identified several alterations in the KO cortex compared to WT, including changes in synapse organization, calcium ion homeostasis, metal ion transport regulation, neuronal projection development, and hypoxia response. They also found impairments in mitochondrial organization, transport, and oxidative phosphorylation (OXPHOS) pathways. Importantly, rhNGF treatment had a positive effect on mitochondrial function and OXPHOS-related pathways. While untreated KO tissues exhibited lower ATP production in their mitochondria, no such defect was found in mitochondria from treated KO mice, highlighting the drug’s beneficial impact.

These findings were further supported by electron microscopy, which measured mitochondrial size and cristae integrity. In untreated KO cortices, mitochondria showed abnormal cristae morphology and increased size, indicative of swelling. However, rhNGF treatment restored normal organelle size and significantly improved cristae organization. This result strengthens the hypothesis that Rett syndrome is associated with altered mitochondrial function and elevated cellular oxidative stress, both of which could significantly contribute to the disease pathogenesis (Shulyakova et al., 2017). Interestingly, some studies suggest that NGF receptors may also be expressed in mitochondrial membranes and that NGF itself could have a protective role in rat brain mitochondria (Carito et al., 2012). These findings raise the possibility of a direct effect of rhNGF on mitochondria in RTT, though further investigation is needed to confirm this hypothesis.

The study on hNGFp conducted by Tiberi and colleagues, instead, focused its attention on the inflammatory aspects of the disease, with particular attention to microglial cells, because previous findings of the group demonstrated that microglia are a TrkA-dependent non-neuronal target cell 535 population of NGF in the brain (Capsoni et al., 2017; Rizzi et al., 2018; Tiberi et al., 2022). These cells are the resident immune cells of the brain, and play a crucial role in immune defense (Borst et al., 2021; Paolicelli et al., 2022). Indeed, their functions extend beyond pathogen response to include the attempted regulation of neurodevelopmental disorders and other brain diseases. While some debate has been addressed (Derecki et al., 2012; Wang et al., 2015), it is now widely accepted that microglia contribute significantly to disease progression (Schafer et al., 2016). In this framework, their research revealed a deficit in microglial morphology in heterozygous female mice, which was completely reversed following hNGFp treatment. This effect on microglia aligns with previous findings (Capsoni et al., 2017; Rizzi et al., 2018; Tiberi et al., 2022). Moreover, they demonstrated that hNGFp restored the altered expression of key neuroimmune communication molecules, such as fractalkine (CX3CL1). Lastly, an *in vitro* study showed that microglia lacking MECP2 exhibit a more aggressive pruning of neuronal spines, a phenomenon that was mitigated by hNGFp treatment. Overall, their findings suggest that hNGFp exerts a protective effect by restoring microglial homeostasis, normalizing their phenotype and function, and ultimately limiting aberrant neuroimmune interactions that contribute to disease pathology.

Taken together, these results offer a compelling insight. The analysis on mitochondria by Pozzer and colleagues provides valuable physiological understanding. Since it does not specify which types of cortical cells exhibit these mitochondrial deficits in KO mice, nor which cells are restored by NGF treatment, it is plausible that mitochondrial dysfunction could affect different cell types in the cortical area, thus both neurons and glia, including microglia. Moreover, mitochondrial dysfunction in microglia has been specifically investigated in Rett syndrome. A decade ago, molecular mechanisms were identified linking MeCP2 to the regulation of bioenergetic pathways in microglia (Jin et al., 2015). Furthermore, transcriptomic studies have revealed that microglia in Rett syndrome exhibit impaired responses to heat stress and other environmental stressors (Zhao et al., 2017). However, at this stage, it remains premature to conclude that mitochondrial dysfunction and immune alterations are mechanistically linked rather than independent processes, and further targeted studies will be required to disentangle these mechanisms in a cell type-specific manner.

These findings underscore the centrality of mitochondrial dysfunction and microglial dysregulation in the pathophysiology of Rett syndrome. The convergence of evidence from both studies suggests that NGF-based treatments may exert their therapeutic effects through a dual mechanism, by restoring mitochondrial homeostasis and normalizing microglia-related neuroinflammatory responses. Considering that mitochondria are crucial regulators of microglial activation and function, it is reasonable to hypothesize that mitochondrial and immune dysfunctions are not independent phenomena but rather interconnected aspects of the same pathological process. This integrated perspective reinforces the therapeutic relevance of targeting both metabolic and immune components in Rett syndrome.

## 8 Conclusion

**Figure 2:**
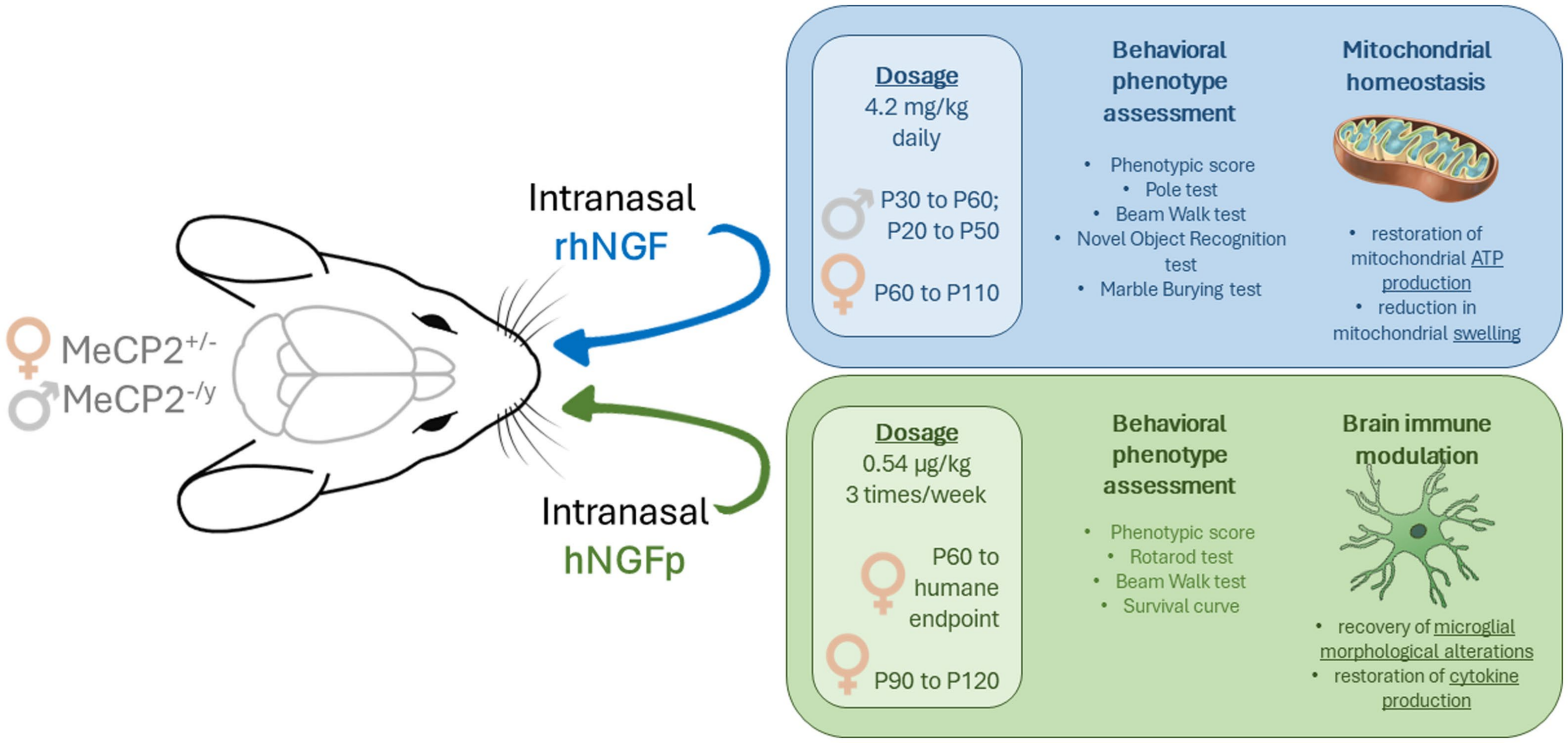
Schematic illustration of intranasal NGF delivery paradigms in Mecp2-deficient mouse models of Rett syndrome. In blue, intranasal administration of rhNGF (4.2 mg/kg daily) in cohorts of male and female Mecp2-deficient mice. These animals underwent both motor and cognitive behavioral assessments, along with evaluation of mitochondrial homeostasis, which was restored in the brains of treated animals. In green, intranasal delivery of hNGFp (0.54 μg/kg, three times per week) in female cohorts, followed by motor behavioral testing. In addition, hNGFp treatment was associated with restoration of microglial phenotype, suggesting a rescue of its abnormal state. Overall, both NGF formulations demonstrated therapeutic efficacy—despite their differences—yielding promising results for the treatment of Rett syndrome.

The two studies reviewed here underscore the therapeutic potential of intranasal NGF-based treatments for Rett syndrome, highlighting both rhNGF and hNGFp as promising candidates, but also noting that the effects of hNGFp were obtained with 8000 fold lower doses. It is tempting to suggest that the lack of p75NTR engagement by the hNGFp protein might underlie its potency at very low doses, consistently found also in previous studies (Capsoni et al., 2017). The ability of NGF and NGF variants molecules to reach the brain via non-invasive intranasal administration represents a significant advancement in the field, overcoming challenges associated with blood–brain barrier permeability and systemic side effects. Both molecules demonstrated neuroprotective effects in RTT mouse models, with rhNGF primarily improving mitochondrial function and cellular respiration, while hNGFp played a key role in restoring neuroinflammation through microglial regulation (Figure 2). However, an important limitation of these studies is the lack of a quantitative assessment of neurotrophin receptor activation, both in terms of the immediate response following administration and the sustained activation induced by repeated dosing, which limits our understanding of the temporal dynamics and persistence of NGF signaling in the brain.

Despite this limitation, the intriguing connection between these two studies lies in the emerging interplay between mitochondrial function and microglial activity in the pathogenesis of Rett syndrome. This convergence suggests that microglia may not only be passive responders to metabolic alterations but active participants in shaping the neurodevelopmental environment through mitochondrial dysfunction. Future investigations into this axis could provide a deeper understanding of how neurotrophic factors like NGF influence cellular energy homeostasis and immune regulation within the brain. In this context, identifying which brain cell types are directly targeted by NGF-based treatments, and in which populations mitochondrial deficits are effectively rescued, will be essential to clarify whether these processes are mechanistically linked or occur in parallel. Despite differences in their molecular mechanisms and dosage, rhNGF and hNGFp yielded behavioral, motor, and cognitive benefits, reinforcing the idea that NGF signaling is a viable therapeutic target for RTT. Notably, the consistency of results across different experimental approaches strengthens the translational potential of NGF-based strategies, although future studies could further expand behavioral assessments by incorporating a broader range of motor and cognitive tests, such as gait analysis for motor coordination, as well as Morris water maze and fear conditioning to more comprehensively evaluate learning and memory behaviors.

One of the most compelling insights from these studies is that early and prolonged treatment (preventive) appears to be more effective than short-term, curative interventions. This aligns with the concept that maintaining NGF levels in the brain fosters a neuroprotective and glioprotective environment, potentially mitigating disease progression rather than merely alleviating symptoms.

However, several aspects warrant further investigation. Future studies should refine dosing strategies, explore sex-dependent responses more thoroughly, and directly compare the long-term safety and efficacy of rhNGF and hNGFp in larger cohorts. Moreover, while both molecules show promise, their differential mechanisms of action raise important questions about their optimal clinical application.

In conclusion, the consistent findings from these studies provide a solid foundation for advancing NGF-based therapies for RTT. Further research will be pivotal in refining treatment protocols and facilitating their effective translation into clinical practice, ultimately encouraging optimism for improved outcomes in RTT patients.

## Footnotes

1
https://www.daybue.com/

## References

[ref1] Aloe L. Rocco M. L. Bianchi P. Manni L. (2012). Nerve growth factor: from the early discoveries to the potential clinical use. J. Transl. Med. 10, 1–15. doi: 10.1186/1479-5876-10-239, 23190582 PMC3543237

[ref2] Amir R. E. Van Den Veyver I. B. Wan M. Tran C. Q. Francke U. Zoghbi H. Y. (1999). Rett syndrome is caused by mutations in X-linked MECP2, encoding methyl- CpG-binding protein 2. Nat. Genet. 23, 185–188. doi: 10.1038/13810, 10508514

[ref3] Anand P. Terenghi G. Warner G. Kopelman P. Williams-Chestnut R. E. Sinicropi D. V. (1996). The role of endogenous nerve growth factor in human diabetic neuropathy. Nat. Med. 2, 703–707. doi: 10.1038/nm0696-703, 8640566

[ref4] Anderson A. Wong K. Jacoby P. Downs J. Leonard H. (2014). Twenty years of surveillance in Rett syndrome: what does this tell us? Orphanet J. Rare Dis. 9, 1–9. doi: 10.1186/1750-1172-9-87, 24942262 PMC4078387

[ref5] Apfel S. C. (2002). Nerve growth factor for the treatment of diabetic neuropathy: what went wrong, what went right, and what does the future hold? Int. Rev. Neurobiol. 50, 393–413. doi: 10.1016/s0074-7742(02)50083-0 12198818

[ref6] Apfel C. C. Kranke P. Eberhart L. H. J. Roos A. Roewer N. (2002). Comparison of predictive models for postoperative nausea and vomiting. Br. J. Anaesth. 88, 234–240. doi: 10.1093/bja/88.2.234, 11883387

[ref7] Apfel S. C. Schwartz S. Adornato B. T Freeman R. Biton V. Rendell M. . (2000). Efficacy and safety of recombinant human nerve growth factor in patients with diabetic polyneuropathy: A randomized controlled trial. rhNGF Clinical Investigator Group. JAMA 284, 2215–2221. doi: 10.1001/jama.284.17.2215 11056593

[ref8] Baker A. M. Batchelor D. C. Thomas G. B. Wen J. Y. Rafiee M. Lin H. . (2005). Central penetration and stability of N-terminal tripeptide of insulin-like growth factor-I, glycine-proline-glutamate in adult rat. Neuropeptides 39, 81–87. doi: 10.1016/j.npep.2004.11.001, 15752541

[ref9] Ballinger E. C. Schaaf C. P. Patel A. J. de Maio A. Tao H. Talmage D. A. . (2019). Mecp2 deletion from cholinergic neurons selectively impairs recognition memory and disrupts cholinergic modulation of the perirhinal cortex. eNeuro 6, 1–13. doi: 10.1523/ENEURO.0134-19.2019 PMC6825959 31562178

[ref10] Berger-Sweeney J. (2011). Cognitive deficits in Rett syndrome: what we know and what we need to know to treat them. Neurobiol. Learn. Mem. 96, 637–646. doi: 10.1016/j.nlm.2011.05.006, 21624480

[ref11] Bonomini F. Favero G. Castrezzati S. Borsani E. (2023). Role of neurotrophins in orofacial pain modulation: a review of the latest discoveries. Int. J. Mol. Sci. 24:12438. doi: 10.3390/ijms241512438, 37569811 PMC10419393

[ref12] Borst K. Dumas A. A. Prinz M. (2021). Microglia: immune and non-immune functions. Immunity 54, 2194–2208. doi: 10.1016/j.immuni.2021.09.014 34644556

[ref13] Brunetti S. Lumsden D. E. (2020). Rett syndrome as a movement and motor disorder – a narrative review. Eur. J. Paediatric Neurol. 28, 29–37. doi: 10.1016/j.ejpn.2020.06.020, 32807681

[ref14] Byiers B. J. Merbler A. M. Raiter A. Burkitt C. C. Symons F. J. (2023). Caregiver perspectives on pain sensitivity and pain experience in Rett syndrome. Can. J. Pain 7:2229400. doi: 10.1080/24740527.2023.2229400, 37533505 PMC10392763

[ref15] Cameron H. A. Hazel T. G. McKay R. D. (1998). Regulation of neurogenesis by growth factors and neurotransmitters. J. Neurobiol. 36, 287–306. doi: 10.1002/(sici)1097-4695(199808)36:2<>3.0.co;2-b, 9712310

[ref16] Capsoni S. Covaceuszach S. Marinelli S. Ceci M. Bernardo A. Minghetti L. . (2011). Taking pain out of NGF: a ‘painless’ NGF mutant, linked to hereditary sensory autonomic neuropathy type V, with full neurotrophic activity. PLoS One 6:e17321. doi: 10.1371/journal.pone.0017321, 21387003 PMC3046150

[ref17] Capsoni S. Malerba F. Carucci N. M. Rizzi C. Criscuolo C. Origlia N. . (2017). The chemokine CXCL12 mediates the anti-amyloidogenic action of painless human nerve growth factor. Brain 140, 201–217. doi: 10.1093/brain/aww271, 28031222 PMC5379860

[ref18] Capsoni S. Marinelli S. Ceci M. Vignone D. Amato G. Malerba F. . (2012). Intranasal ‘painless’ human nerve growth factors slows amyloid neurodegeneration and prevents memory deficits in app X PS1 mice. PLoS One 7:7555. doi: 10.1371/journal.pone.0037555, 22666365 PMC3364340

[ref19] Carito V. Pingitore A. Cione E. Perrotta I. Mancuso D. Russo A. . (2012). Localization of nerve growth factor (NGF) receptors in the mitochondrial compartment: characterization and putative role. Biochim. Biophys. Acta 1820, 96–103. doi: 10.1016/j.bbagen.2011.10.015, 22138126

[ref20] Carvalho A. L. Caldeira M. V. Santos S. D. Duarte C. B. (2008). Role of the brain-derived neurotrophic factor at glutamatergic synapses: brain-derived neurotrophic factor. Br. J. Pharmacol. 153, S310–S324. doi: 10.1038/sj.bjp.0707509 18059328 PMC2268077

[ref21] Castro J. Garcia R. I. Kwok S. Banerjee A. Petravicz J. Woodson J. . (2014). Functional recovery with recombinant human IGF1 treatment in a mouse model of Rett syndrome. Proc. Natl. Acad. Sci. USA 111, 9941–9946. doi: 10.1073/pnas.1311685111, 24958891 PMC4103342

[ref22] Chahrour M. Jung S. Y. Shaw C. Zhou X. Wong S. T. C. Qin J. . (2008). MeCP2, a key contributor to neurological disease, activates and represses transcription. Science 320, 1224–1229. doi: 10.1126/science.1153252, 18511691 PMC2443785

[ref23] Chang Q. Khare G. Dani V. Nelson S. Jaenisch R. (2006). The disease progression of Mecp2 mutant mice is affected by the level of BDNF expression. Neuron 49, 341–348. doi: 10.1016/j.neuron.2005.12.027, 16446138

[ref24] Chen W. G. Chang Q. Lin Y. Meissner A. West A. E. Griffith E. C. . (2003). Derepression of BDNF transcription involves calcium-dependent phosphorylation of MeCP2. Science 302, 885–889. doi: 10.1126/science.1086446 14593183

[ref25] Chiaretti A. Conti G. Falsini B. Buonsenso D. Crasti M. Manni L. . (2017). Intranasal nerve growth factor administration improves cerebral functions in a child with severe traumatic brain injury: a case report. Brain Inj. 31, 1538–1547. doi: 10.1080/02699052.2017.1376760, 28972396

[ref26] Chiricozzi E. Di Biase E. Maggioni M. Biase E. D. Lunghi G. Fazzari M. . (2019). GM1 promotes TrkA-mediated neuroblastoma cell differentiation by occupying a plasma membrane domain different from TrkA. J. Neurochem. 149, 231–241. doi: 10.1111/jnc.14685, 30776097

[ref27] Chiricozzi E. Pomè D. Y. Maggioni M. di Biase E. Parravicini C. Palazzolo L. . (2017). Role of the GM1 ganglioside oligosaccharide portion in the TrkA-dependent neurite sprouting in neuroblastoma cells. J. Neurochem. 143, 645–659. doi: 10.1111/jnc.14146, 28796418

[ref28] Chuang H. H. Prescott E. D. Kong H. Chuang H. Shields S. Jordt S.-E. . (2001). Bradykinin and nerve growth factor release the capsaicin receptor from PtdIns(4,5)P2-mediated inhibition. Nature 411, 957–962. doi: 10.1038/35082088, 11418861

[ref001] Covaceuszach S. Capsoni S. Marinelli S. Pavone F. Ceci M. Ugolini G. . (2010). In vitro receptor binding properties of a “painless” NGF mutein, linked to hereditary sensory autonomic neuropathy type V. Biochem Biophys Res Commun. 391, 824–9. doi: 10.1016/j.bbrc.2009.11.146, 19945432

[ref29] Curatola A. Graglia B. Granata G. Conti G. Capossela L. Manni L. . (2023). Combined treatment of nerve growth factor and transcranical direct current stimulations to improve outcome in children with vegetative state after out-of-hospital cardiac arrest. Biol. Direct 18:24. doi: 10.1186/s13062-023-00379-5, 37165387 PMC10170696

[ref30] Deogracias R. Yazdani M. Dekkers M. P. J. Guy J. Ionescu M. C. S. Vogt K. E. . (2012). Fingolimod, a Sphingosine-1 phosphate receptor modulator, increases BDNF levels and improves symptoms of a mouse model of Rett syndrome. Proc. Natl. Acad. Sci. USA 109, 14230–14235. doi: 10.1073/pnas.1206093109, 22891354 PMC3435172

[ref31] Derecki N. C. Cronk J. C. Lu Z. Xu E. Abbott S. B. G. Guyenet P. G. . (2012). Wild-type microglia arrest pathology in a mouse model of Rett syndrome. Nature 484, 105–109. doi: 10.1038/nature10907, 22425995 PMC3321067

[ref32] DiStefano P. S. Friedman B. Radziejewski C. Alexander C. Boland P. Schick C. M. . (1992). The neurotrophins BDNF, NT-3, and NGF display distinct patterns of retrograde axonal transport in peripheral and central neurons. Neuron 8, 983–993. doi: 10.1016/0896-6273(92)90213-W, 1375039

[ref33] Einarsdottir E. Carlsson A. Minde J. Toolanen G. Svensson O. Solders G. . (2004). A mutation in the nerve growth factor beta gene (NGFB) causes loss of pain perception. Hum. Mol. Genet. 13, 799–805. doi: 10.1093/hmg/ddh096 14976160

[ref34] Escamilla-Del-Arenal M. da Rocha S. T. Edith H. (2011). Evolutionary diversity and developmental regulation of X-chromosome inactivation. Hum. Genet. 130, 307–327. doi: 10.1007/s00439-011-1029-2 21687993 PMC3132430

[ref35] Fabio R. A. Chiarini L. Canegallo V. (2022). Pain in Rett syndrome: a pilot study and a single case study on the assessment of pain and the construction of a suitable measuring scale. Orphanet J. Rare Dis. 17:356. doi: 10.1186/s13023-022-02519-y, 36104823 PMC9476284

[ref36] Fazzari M. Lunghi G. Carsana E. V. Valsecchi M. Spiombi E. Breccia M. . (2024). GM1 oligosaccharide ameliorates Rett syndrome phenotypes in vitro and in vivo via Trk receptor activation. Int. J. Mol. Sci. 25:11555. doi: 10.3390/ijms252111555, 39519108 PMC11547101

[ref37] Fiore M. Chaldakov G. N. Aloe L. (2009). Nerve growth factor as a signaling molecule for nerve cells and also for the neuroendocrine-immune systems. Rev. Neurosci. 20, 133–145. doi: 10.1515/REVNEURO.2009.20.2.133, 19774790

[ref38] Frey W. H. Liu J. Chen X. Thorne R. G. Fawcett J. R. Ala T. A. . (1997). Delivery of ^125^I-NGF to the brain via the olfactory route. Drug Deliv. 4, 87–92. doi: 10.3109/10717549709051878

[ref39] Galvez-Contreras A. Y. Campos-Ordonez T. Gonzalez-Castaneda R. E. Gonzalez-Perez O. (2017). Alterations of growth factors in autism and attention-deficit/hyperactivity disorder. Front. Psych. 8:126. doi: 10.3389/fpsyt.2017.00126 PMC5507945 28751869

[ref40] Galvez-Contreras A. Y. Campos-Ordonez T. Lopez-Virgen V. Gomez-Plascencia J. Ramos-Zuniga R. Gonzalez-Perez O. (2016). Growth factors as clinical biomarkers of prognosis and diagnosis in psychiatric disorders. Cytokine Growth Factor Rev. 32, 85–96. doi: 10.1016/j.cytogfr.2016.08.004 27618303

[ref41] Gatto A. Capossela L. Conti G. Eftimiadi G. Ferretti S. Manni L. . (2023). Intranasal human-recombinant NGF administration improves outcome in children with post-traumatic unresponsive wakefulness syndrome. Biol. Direct 18:61. doi: 10.1186/s13062-023-00418-1, 37789391 PMC10546699

[ref42] Gomez D. Martinez J. A. Hanson L. R. Frey W. H. 2nd Toth C. C. (2012). Intranasal treatment of neurodegenerative diseases and stroke. Front. Biosci. (Schol. Ed.) 4, 74–89. doi: 10.2741/s252 22202044

[ref43] Guideri F. Acampa M. Calamandrei G. Aloe L. Zappella M. Hayek Y. (2004). Nerve growth factor plasma levels and ventricular repolarization in Rett syndrome. Pediatr. Cardiol. 25, 394–396. doi: 10.1007/s00246-002-0406-y 14708067

[ref44] Guy J. Gan J. Selfridge J. Cobb S. Bird A. (2007). Reversal of neurological defects in a mouse model of Rett syndrome. Science 315, 1143–1147. doi: 10.1126/science.1138389, 17289941 PMC7610836

[ref45] Guy J. Hendrich B. Holmes M. Martin J. E. Bird A. (2001). A mouse Mecp2-null mutation causes neurological symptoms that mimic Rett syndrome. Nat. Genet. 27, 322–326. doi: 10.1038/85899, 11242117

[ref46] Hagberg B. Aicardi J. Dias K. Ramos O. (1983). A progressive syndrome of autism, dementia, Ataxia, and loss of purposeful hand use in girls: Rett’s syndrome: report of 35 cases. Ann. Neurol. 14, 471–479. doi: 10.1002/ana.410140412, 6638958

[ref47] Hefti F. (1994). Development of effective therapy for Alzheimer’s disease based on neurotrophic factors. Neurobiol. Aging 15, S193–S194. doi: 10.1016/0197-4580(94)90204-6 7700452

[ref48] Hudu S. A. Elmigdadi F. Al Qtaitat A. Qtaitat A. A. Almehmadi M. Alsaiari A. A. . (2023). Trofinetide for Rett syndrome: highlights on the development and related inventions of the first USFDA-approved treatment for rare pediatric unmet medical need. J. Clin. Med. 12:5114. doi: 10.3390/jcm12155114, 37568516 PMC10420089

[ref49] Jin L.-W. Horiuchi M. Wulff H. Liu X.-B. Cortopassi G. A. Erickson J. D. . (2015). Dysregulation of glutamine transporter SNAT1 in Rett syndrome microglia: a mechanism for mitochondrial dysfunction and neurotoxicity. J. Neurosci. Off. J. Soc. Neurosci. 35, 2516–2529. doi: 10.1523/JNEUROSCI.2778-14.2015, 25673846 PMC4323531

[ref50] Katz D. M. Berger-Sweeney J. E. Eubanks J. H. Justice M. J. Neul J. L. Pozzo-Miller L. . (2012). Preclinical research in Rett syndrome: setting the Foundation for Translational Success. Dis. Model. Mech. 5, 733–745. doi: 10.1242/dmm.011007, 23115203 PMC3484856

[ref51] Keam S. J. (2023). Trofinetide: first approval. Drugs 83, 819–824. doi: 10.1007/s40265-023-01883-8, 37191913

[ref52] Kraemer B. R. Snow J. P. Vollbrecht P. Pathak A. Valentine W. M. Deutch A. Y. . (2014). A Role for the p75 neurotrophin receptor in axonal degeneration and apoptosis induced by oxidative stress. J. Biol. Chem. 289, 21205–21216. doi: 10.1074/jbc.M114.563403, 24939843 PMC4118083

[ref53] Kumar N. N. Gautam M. Lochhead J. J. Wolak D. J. Ithapu V. Singh V. . (2016). Relative vascular permeability and vascularity across different regions of the rat nasal mucosa: implications for nasal physiology and drug delivery. Sci. Rep. 6:31732. doi: 10.1038/srep31732, 27558973 PMC4997340

[ref54] Lappalainen R. Lindholm D. Riikonen R. (1996). Low levels of nerve growth factor in cerebrospinal fluid of children with Rett syndrome. J. Child Neurol. 11, 296–300. doi: 10.1177/088307389601100407, 8807419

[ref55] Ledeen R. W. Wu G. (2008). Thematic review series: sphingolipids. Nuclear sphingolipids: metabolism and signaling. J. Lipid Res. 49, 1176–1186. doi: 10.1194/jlr.R800009-JLR200, 18326892 PMC2386903

[ref56] Lekman A. Y. Hagberg B. A. Svennerholm L. T. (1991). Membrane cerebral lipids in Rett syndrome. Pediatr. Neurol. 7, 186–190. doi: 10.1016/0887-8994(91)90082-v, 1878098

[ref57] Lekman A. Y. Hagberg B. A. Svennerholm L. T. (1999). Cerebrospinal fluid gangliosides in patients with Rett syndrome and infantile neuronal ceroid lipofuscinosis. Eur. J. Paediatric Neurol. 3, 119–123. doi: 10.1016/S1090-3798(99)90099-5, 10461567

[ref58] Levi-Montalcini R. (1952). Effects of mouse tumor transplantation on the nervous system. Ann. N. Y. Acad. Sci. 55, 330–344. doi: 10.1111/j.1749-6632.1952.tb26548.x, 12977049

[ref59] Levi-Montalcini R. (1966). The nerve growth factor: its mode of action on sensory and sympathetic nerve cells. Harvey Lect. 60, 217–259. 5338067

[ref60] Lewin G. R. Mendell L. M. (1993). Nerve growth factor and nociception. Trends Neurosci. 16, 353–359. doi: 10.1016/0166-2236(93)90092-z, 7694405

[ref61] Lewin G. R. Ritter A. M. Mendell L. M. (1993). Nerve growth factor-induced hyperalgesia in the neonatal and adult rat. J. Neurosci. 13, 2136–2148. doi: 10.1523/JNEUROSCI.13-05-02136.1993, 8478693 PMC6576576

[ref62] Lipani J. D. Bhattacharjee M. B. Corey D. M. Lee D. A. (2000). Reduced nerve growth factor in Rett syndrome postmortem brain tissue. J. Neuropathol. Exp. Neurol. 59, 889–895. doi: 10.1093/jnen/59.10.889, 11079779

[ref63] Lockhart S. T. Turrigiano G. G. Birren S. J. (1997). Nerve growth factor modulates synaptic transmission between sympathetic neurons and cardiac myocytes. J. Neurosci. Off. J. Soc. Neurosci. 17, 9573–9582. doi: 10.1523/JNEUROSCI.17-24-09573.1997, 9391012 PMC6573427

[ref64] Malerba F. Paoletti F. Bruni Ercole B. Materazzi S. Nassini R. Coppi E. . (2015). Functional characterization of human proNGF and NGF mutants: identification of NGF P61SR100E as a ‘painless’ lead investigational candidate for therapeutic applications. PLoS One 10:e0136425. doi: 10.1371/journal.pone.0136425 26371475 PMC4570711

[ref65] Malerba F. Paoletti F. Capsoni S. Cattaneo A. (2011). Intranasal delivery of therapeutic proteins for neurological diseases. Expert Opin. Drug Deliv. 8, 1277–1296. doi: 10.1517/17425247.2011.588204, 21619468

[ref66] Manni L. Conti G. Chiaretti A. Soligo M. (2021). Intranasal delivery of nerve growth factor in neurodegenerative diseases and Neurotrauma. Front. Pharmacol. 12:754502. doi: 10.3389/fphar.2021.754502, 34867367 PMC8635100

[ref67] Massa S. M. Xie Y. Yang T. Harrington A. W. Kim M. L. Yoon S. O. . (2006). Small, nonpeptide p75NTR ligands induce survival signaling and inhibit proNGF-induced death. J. Neurosci. Off. J. Soc. Neurosci. 26, 5288–5300. doi: 10.1523/JNEUROSCI.3547-05.2006, 16707781 PMC6675309

[ref68] McArthur J. C. Yiannoutsos C. Simpson D. M. Adornato B. T. Singer E. J. Hollander H. . (2000). A phase II trial of nerve growth factor for sensory neuropathy associated with HIV infection. Neurology 54, 1080–1088. doi: 10.1212/wnl.54.5.1080 10720278

[ref69] Mufson E. J. Kroin J. S. Sendera T. J. Sobreviela T. (1999). Distribution and retrograde transport of trophic factors in the central nervous system: functional implications for the treatment of neurodegenerative diseases. Prog. Neurobiol. 57, 451–484. doi: 10.1016/S0301-0082(98)00059-8, 10080385

[ref70] Naegelin Y. Kuhle J. Schädelin S. Datta A. N. Magon S. Amann M. . (2021). Fingolimod in children with Rett syndrome: the FINGORETT study. Orphanet J. Rare Dis. 16:19. doi: 10.1186/s13023-020-01655-7, 33407685 PMC7789265

[ref71] Neul J. L. Benke T. A. Marsh E. D. Skinner S. A. Merritt J. Lieberman D. N. . (2019). The array of clinical phenotypes of males with mutations in methyl-CpG binding protein 2. Am. J. Med. Genet. B Neuropsychiatr. Genet. 180, 55–67. doi: 10.1002/ajmg.b.32707, 30536762 PMC6488031

[ref72] Neul J. L. Zoghbi H. Y. (2004). Rett syndrome: a prototypical neurodevelopmental disorder. Neuroscientist 10, 118–128. doi: 10.1177/1073858403260995, 15070486

[ref73] Nomura Y. Segawa M. (1992). Motor symptoms of the Rett syndrome: abnormal muscle tone, posture, locomotion and stereotyped movement. Brain Dev. 14, S21–S28. 1626630

[ref74] O’Kusky J. Ye P. (2012). Neurodevelopmental effects of insulin-like growth factor signaling. Front. Neuroendocrinol. 33, 230–251. doi: 10.1016/j.yfrne.2012.06.002, 22710100 PMC3677055

[ref75] O’Leary H. M. Marschik P. B. Khwaja O. S. Ho E. Barnes K. V. Clarkson T. W. . (2017). Detecting autonomic response to pain in Rett syndrome. Dev. Neurorehabil. 20, 108–114. doi: 10.3109/17518423.2015.1087437, 26457613

[ref76] Ogier M. Wang H. Hong E. Wang Q. Greenberg M. E. Katz D. M. (2007). Brain-derived neurotrophic factor expression and respiratory function improve after Ampakine treatment in a mouse model of Rett syndrome. J. Neurosci. Off. J. Soc. Neurosci. 27, 10912–10917. doi: 10.1523/JNEUROSCI.1869-07.2007, 17913925 PMC6672830

[ref77] Pan W. Banks W. A. Kastin A. J. (1998). Permeability of the blood-brain barrier to neurotrophins. Brain Res. 788, 87–94. doi: 10.1016/s0006-8993(97)01525-4, 9554964

[ref78] Paolicelli R. C. Sierra A. Stevens B. Tremblay M.-E. Aguzzi A. Ajami B. . (2022). Microglia states and nomenclature: a field at its crossroads. Neuron 110, 3458–3483. doi: 10.1016/j.neuron.2022.10.020, 36327895 PMC9999291

[ref79] Perry E. K. (1990). Nerve growth factor and the basal forebrain cholinergic system: a link in the etiopathology of neurodegenerative dementias? Alzheimer Dis. Assoc. Disord. 4, 1–13. doi: 10.1097/00002093-199040100-00001, 2180439

[ref80] Petty B. G. Cornblath D. R. Adornato B. T. Chaudhry V. Flexner C. Wachsman M. . (1994). The effect of systemically administered recombinant human nerve growth factor in healthy human subjects. Ann. Neurol. 36, 244–246. doi: 10.1002/ana.410360221, 8053664

[ref81] Pitto M. Mutoh T. Kuriyama M. Ferraretto A. Palestini P. Masserini M. (1998). Influence of endogenous GM1 ganglioside on TrkB activity, in cultured neurons. FEBS Lett. 439, 93–96. doi: 10.1016/s0014-5793(98)01344-1, 9849885

[ref82] Poduslo J. F. Curran G. L. (1996). Permeability at the blood-brain and blood-nerve barriers of the neurotrophic factors: NGF, CNTF, NT-3, BDNF. Mol. Brain Res. 36, 280–286. doi: 10.1016/0169-328x(95)00250-v, 8965648

[ref83] Pozzer D. Indrigo M. Breccia M. Florio E. Franchino C. A. De Rocco G. . (2025). Clinical-grade intranasal NGF fuels neurological and metabolic functions of Mecp2-deficient mice. Brain 148, 845–860. doi: 10.1093/brain/awae291, 39300821 PMC11884770

[ref84] Reichard J. Zimmer-Bensch G. (2021). The epigenome in neurodevelopmental disorders. Front. Neurosci. 15:776809. doi: 10.3389/fnins.2021.776809, 34803599 PMC8595945

[ref85] Ribeiro M. C. MacDonald J. L. (2020). Sex differences in Mecp2-mutant Rett syndrome model mice and the impact of cellular mosaicism in phenotype development. Brain Res. 1729:146644. doi: 10.1016/j.brainres.2019.146644, 31904347 PMC7024565

[ref86] Rizzi C. Tiberi A. Giustizieri M. Marrone M. C. Gobbo F. Carucci N. M. . (2018). NGF steers microglia toward a neuroprotective phenotype. Glia 66, 1395–1416. doi: 10.1002/glia.23312, 29473218 PMC6001573

[ref87] Rosa D. Garcia R. Braschi A. A. (2005). Intranasal administration of nerve growth factor (NGF) rescues recognition memory deficits in AD11 anti-NGF transgenic mice. Proc. Natl. Acad. Sci. USA 102, 3811–3816. doi: 10.1073/pnas.0500195102, 15728733 PMC553297

[ref88] Roszkowska A. M. Spinella R. Calderone A. Sindoni M. Wowra B. H. Kozak M. . (2024). The use of Rh-NGF in the management of neurotrophic keratopathy. Front. Ophthalmol. 4:1408587. doi: 10.3389/fopht.2024.1408587, 39040985 PMC11260816

[ref89] Sacchetti M. Lambiase A. Schmidl D. Schmetterer L. Ferrari M. Mantelli F. . (2020). Effect of recombinant human nerve growth factor eye drops in patients with dry eye: a phase IIa, open label, multiple-dose study. Br. J. Ophthalmol. 104, 127–135. doi: 10.1136/bjophthalmol-2018-312470 30944103 PMC6922013

[ref90] Schafer D. P. Heller C. T. Gunner G. Heller M. Gordon C. Hammond T. . (2016). Microglia contribute to circuit defects in Mecp2 null mice independent of microglia-specific loss of Mecp2 expression. eLife 5:e15224. doi: 10.7554/elife.15224 27458802 PMC4961457

[ref91] Schmid D. A. Yang T. Ogier M. Adams I. Mirakhur Y. Wang Q. . (2012). A TrkB small molecule partial agonist rescues TrkB phosphorylation deficits and improves respiratory function in a mouse model of Rett syndrome. J. Neurosci. Off. J. Soc. Neurosci. 32, 1803–1810. doi: 10.1523/JNEUROSCI.0865-11.2012, 22302819 PMC3710112

[ref92] Shanks H. R. C. Chen K. Reiman E. M. Blennow K. Cummings J. L. Massa S. M. . (2024). p75 neurotrophin receptor modulation in mild to moderate Alzheimer disease: a randomized, placebo-controlled phase 2a trial. Nat. Med. 30, 1761–1770. doi: 10.1038/s41591-024-02977-w, 38760589 PMC11186782

[ref93] Shinoda M. Asano M. Omagari D. Honda K. Hitomi S. Katagiri A. . (2011). Nerve growth factor contribution via transient receptor potential vanilloid 1 to ectopic orofacial pain. J. Neurosci. 31, 7145–7155. doi: 10.1523/JNEUROSCI.0481-11.2011 21562277 PMC6703199

[ref94] Shulyakova N. Andreazza A. C. Mills L. R. Eubanks J. H. (2017). Mitochondrial dysfunction in the pathogenesis of Rett syndrome: implications for mitochondria-targeted therapies. Front. Cell. Neurosci. 11:58. doi: 10.3389/fncel.2017.00058 28352216 PMC5348512

[ref95] Strauss S. Otten U. Joggerst B. Pluss K. Volk B. (1994). Increased levels of NGF protein and mRNA and reactive gliosis following Kainic acid injection into the rat striatum. Neurosci. Lett. 168, 193–196. doi: 10.1016/0304-3940(94)90448-0, 8028775

[ref96] Sultana R. Ogundele O. M. Lee C. C. (2019). Contrasting characteristic Behaviours among common laboratory mouse strains. R. Soc. Open Sci. 6:190574. doi: 10.1098/rsos.190574, 31312505 PMC6599779

[ref97] Symons F. J. Byiers B. Tervo R. C. Beisang A. (2013). Parent-reported pain in Rett syndrome. Clin. J. Pain 29, 744–746. doi: 10.1097/AJP.0b013e318274b6bd, 23835769 PMC3707000

[ref98] Thorne R. G. Emory C. R. Ala T. A. Frey W. H. 2nd (1995). Quantitative analysis of the olfactory pathway for drug delivery to the brain. Brain Res. 692, 278–282. doi: 10.1016/0006-8993(95)00637-6, 8548316

[ref99] Thorne R. G. Frey W. H. (2001). Delivery of neurotrophic factors to the central nervous system. Clin. Pharmacokinet. 40, 907–946. doi: 10.2165/00003088-200140120-00003, 11735609

[ref100] Tiberi A. Borgonovo G. Testa G. Pacifico P. Jacob A. Di Caprio M. . (2024). Reversal of neurological deficits by painless nerve growth factor in a mouse model of Rett syndrome. Brain 147, 122–134. doi: 10.1093/brain/awad282, 37633263 PMC10766238

[ref101] Tiberi A. Capsoni S. Cattaneo A. (2022). A microglial function for the nerve growth factor: predictions of the unpredictable. Cells 11:1835. doi: 10.3390/cells11111835, 35681529 PMC9180430

[ref103] Varone M. Scavo G. Colardo M. Martella N. Pensabene D. Bisesto E. . (2024). P75NTR modulation reduces oxidative stress and the expression of pro-inflammatory mediators in a cell model of Rett syndrome. Biomedicine 12:2624. doi: 10.3390/biomedicines12112624, 39595188 PMC11592079

[ref104] Wang J. Wegener J. E. Huang T.-W. Sripathy S. de Jesus-Cortes H. Xu P. . (2015). Wild-type microglia do not reverse pathology in mouse models of Rett syndrome. Nature 521, E1–E4. doi: 10.1038/nature14444, 25993969 PMC4684952

[ref105] Watanabe T. Ito T. Inoue G. Ohtori S. Kitajo K. Doya H. . (2008). The p75 receptor is associated with inflammatory thermal hypersensitivity. J. Neurosci. Res. 86, 3566–3574. doi: 10.1002/jnr.21808, 18709654

[ref106] Wenk G. L. Hauss-Wegrzyniak B. (1999). Altered cholinergic function in the basal forebrain of girls with Rett syndrome. Neuropediatrics 30, 125–129. doi: 10.1055/s-2007-973476, 10480206

[ref107] Wenk G. L. O’Leary M. Nemeroff C. B. Bissette G. Moser H. Naidu S. (1993). Neurochemical alterations in Rett syndrome. Brain Res. Dev. Brain Res. 74, 67–72. doi: 10.1016/0165-3806(93)90084-N, 8403377

[ref108] Yang C. Chapman A. G. Kelsey A. D. Minks J. Cotton A. M. Brown C. J. (2011). X-chromosome inactivation: molecular mechanisms from the human perspective. Hum. Genet. 130, 175–185. doi: 10.1007/s00439-011-0994-9, 21553122

[ref109] Zhao D. Mokhtari R. Pedrosa E. Birnbaum R. Zheng D. Lachman H. M. (2017). Transcriptome analysis of microglia in a mouse model of Rett syndrome: differential expression of genes associated with microglia/macrophage activation and cellular stress. Mol. Autism. 8:17. doi: 10.1186/s13229-017-0134-z 28367307 PMC5372344

[ref110] Zhou H. Wu W. Zhang Y. He H. Yuan Z. Zhu Z. . (2017). Selective preservation of cholinergic MeCP2 rescues specific Rett-syndrome-like phenotypes in MeCP2 stop mice. Behav. Brain Res. 322, 51–59. doi: 10.1016/j.bbr.2017.01.023, 28093257

